# An umbrella protocol for the clinical evaluation of diagnostics in infectious disease

**DOI:** 10.2471/BLT.25.293246

**Published:** 2026-04-29

**Authors:** Edward Blandford, Sarah Tunkel, Elizabeth Coates, Carolina R Arevalo, Simon E Kolstoe, Obaghe Edeghere, Fiona Maleady-Crowe, David Chapman, Carolyn Lewis, Raghavendran Kulasegaran-Shylini, Richard Vipond, Deborah A Williamson, Richard Pebody, Isabel Oliver, Susan Hopkins, Sue Hill, Tom Fowler

**Affiliations:** a UK Health Security Agency, 10 South Colonnade, Canary Wharf, London, E14 4PU, England.; b School of Health & Care Professions, University of Portsmouth, England.; c Deloitte MCS Ltd, London, England.; d NHS England and NHS Improvement, London, England.

## Abstract

Umbrella protocols have recently come to be widely used in clinical trial designs. However, the value of this approach in public health is less well recognized. The coronavirus disease 2019 (COVID-19) pandemic highlighted the need for rapid, reliable and scalable evaluation of diagnostic technologies. In the United Kingdom of Great Britain and Northern Ireland, this need prompted the development of an umbrella research protocol enabling multiple clinical evaluation studies of similar designs to be undertaken under a single overarching preapproved ethics and governance framework. We describe the development, implementation and evolution of this protocol, which was designed to support timely assessment of the performance of in vitro diagnostic devices and associated testing approaches in various settings. The umbrella protocol allowed studies to be started quickly during the pandemic, reduced administrative burdens, supported regulatory submissions and enabled prospective collection of samples for surveillance. While the system described reflects British governance structures, the principles underpinning this approach, including proportionality (ensuring oversight requirements are appropriate to the risk level), standardization and preapproved flexibility, are applicable to many settings. The protocol now forms part of the United Kingdom’s wider pandemic preparedness structure and illustrates how preapproved, adaptable research frameworks can accelerate evidence generation during outbreaks. The world is now assessing lessons from the COVID-19 pandemic and it is timely to consider how research systems can support innovative designs such as umbrella protocols. We therefore summarize lessons learnt and practical considerations to support other countries seeking to adopt similar approaches within their own ethical and regulatory systems.

## Résumé

De nous jours, les protocoles-cadres sont largement employés dans le design des essais cliniques. Cependant, l’intérêt de cette approche en matière de santé publique est moins bien reconnu. En effet, la pandémie de COVID-19 a mis en évidence la nécessité d’une évaluation rapide, fiable et évolutive des technologies de diagnostic. Au Royaume-Uni, ce besoin a conduit à l’élaboration d’un protocole de recherche global permettant de mener plusieurs études d’évaluation clinique de design similaire dans le cadre d’un cadre global préapprouvé en matière d’éthique et de gouvernance. Nous décrivons l’élaboration, la mise en œuvre et l’évolution de ce protocole, qui a été conçu pour faciliter l’évaluation en temps opportun des performances des dispositifs de diagnostic in vitro et des approches de test associées dans divers contextes. Ce protocole-cadre a permis de lancer rapidement des études pendant la pandémie, d’alléger les charges administratives, de faciliter les demandes d’autorisation réglementaire et de permettre le prélèvement prospectif d’échantillons à des fins de surveillance. Si le système décrit reflète les structures de gouvernance britanniques, les principes qui sous-tendent cette approche, notamment la proportionnalité (garantissant que les exigences en matière de surveillance sont adaptées au niveau de risque), la normalisation et la flexibilité préapprouvée, s’appliquent à de nombreux contextes. Ce protocole fait désormais partie intégrante de la structure plus large du Royaume-Uni dans la préparation aux pandémies et illustre la façon dont des cadres de recherche préapprouvés et adaptables permettent d’accélérer la production de données scientifiques lors d’épidémies. Le monde tire actuellement les leçons de la pandémie de COVID-19 et le moment est venu d’examiner la manière dont les systèmes de recherche sont capables de soutenir des designs innovants tels que les protocoles-cadres. Nous résumons donc les enseignements tirés et les considérations pratiques afin d’aider d’autres pays qui souhaitent adopter des approches similaires au sein de leurs propres systèmes éthiques et réglementaires.

## Resumen

Los protocolos marco han pasado a utilizarse ampliamente en el diseño de ensayos clínicos en los últimos años. Sin embargo, el valor de este enfoque en salud pública es menos reconocido. La pandemia de la enfermedad por coronavirus de 2019 (COVID-19) destacó la necesidad de una evaluación rápida, fiable y escalable de las tecnologías de diagnóstico. En el Reino Unido de Gran Bretaña e Irlanda del Norte, esta necesidad dio lugar al desarrollo de un protocolo de investigación marco que permite llevar a cabo múltiples estudios de evaluación clínica con diseños similares bajo un único marco general de ética y gobernanza previamente aprobado. Se describe el desarrollo, la implementación y la evolución de este protocolo, diseñado para apoyar la evaluación oportuna del rendimiento de dispositivos de diagnóstico in vitro y de los enfoques de prueba asociados en diversos contextos. El protocolo marco permitió iniciar estudios rápidamente durante la pandemia, redujo la carga administrativa, facilitó la presentación ante las autoridades regulatorias y permitió la recogida prospectiva de muestras para la vigilancia. Aunque el sistema descrito refleja las estructuras de gobernanza británicas, los principios que sustentan este enfoque, como la proporcionalidad (asegurar que los requisitos de supervisión sean adecuados al nivel de riesgo), la estandarización y la flexibilidad previamente aprobada, son aplicables en numerosos contextos. El protocolo forma ahora parte de la estructura más amplia de preparación frente a pandemias del Reino Unido y demuestra cómo los marcos de investigación adaptables y previamente aprobados pueden acelerar la generación de evidencia durante brotes. En la actualidad, la comunidad internacional está evaluando las lecciones de la pandemia de la COVID-19, y resulta oportuno considerar cómo los sistemas de investigación pueden apoyar diseños innovadores como los protocolos marco. Por ello, se resumen las lecciones aprendidas y las consideraciones prácticas para apoyar a otros países que deseen adoptar enfoques similares dentro de sus propios sistemas éticos y regulatorios.

## ملخص

أصبح استخدام البروتوكولات الشاملة شائعًا في الآونة الأخيرة في تصميم التجارب الإكلينيكية. مع ذلك، فإن قيمة هذا الأسلوب في الصحة العامة لا تتمتع بالتقدير بالشكل الكافي. إن جائحة مرض فيروس كورونا لعام 2019 (كوفيد 19) قد أوضحت الحاجة إلى تقييم سريع وموثوق وقابل للتطوير لتقنيات التشخيص. في المملكة المتحدة لبريطانيا العظمى وأيرلندا الشمالية، دفعت هذه الحاجة إلى تطوير بروتوكول بحثي شامل يُتيح إجراء دراسات تقييم إكلينيكي متعددة، وذات تصميمات متشابهة، ليتم الاضطلاع بها ضمن إطار عمل أخلاقي وتابع للحوكمة بشكل شامل مُعتمد مسبقًا. في هذا البحث، نحن نقوم بوصف هذا البروتوكول وكيفية تنفيذه وتطويره، والذي تم تصميمه لدعم التقييم في الوقت المناسب لأداء أجهزة التشخيص المختبري، وأساليب الاختبار المرتبطة بها في مختلف الأوضاع. وقد أتاح البروتوكول الشامل بدء الدراسات بسرعة أثناء الجائحة، وحد من الأعباء الإدارية، ودعم تقديم الطلبات التنظيمية، وسمح بجمع العينات بشكل استباقي لأغراض المراقبة. بينما يعكس النظام الموصوف البنى التحتية للحوكمة البريطانية، فإن المبادئ التي يقوم عليها هذا الأسلوب، بما في ذلك التناسب (ضمان ملاءمة متطلبات الرقابة لمستوى المخاطر)، والتوحيد القياسي، والمرونة المعتمدة مسبقًا، تُعد قابلة للتطبيق في العديد من الأوضاع. يشكل البروتوكول الآن جزءًا من البنية التحتية للاستعداد على نطاق أوسع للأوبئة في المملكة المتحدة، ويوضح كيف يمكن لأطر عمل البحث المعتمدة مسبقًا، والقابلة للتكيف، أن تُسرّع من استنتاج الأدلة أثناء تفشي الأوبئة. يشهد العالم الآن تقييمًا للدروس المستفادة من جائحة كوفيد 19، وقد حان الوقت للنظر في كيف يمكن لأنظمة البحث دعم التصاميم المبتكرة مثل البروتوكولات الشاملة. لذلك، نحن نلخص الدروس المستفادة والاعتبارات العملية لدعم الدول الأخرى التي تسعى إلى تبني أساليب مماثلة ضمن أنظمتها الأخلاقية والتنظيمية.

## 摘要

近年来，总括协议已广泛应用于临床试验设计。但是，该方法在公共卫生领域的价值尚未得到广泛认可。2019 冠状病毒病 (COVID-19) 大流行疫情凸显了对诊断技术进行快速、可靠和可扩展评估的需求。这一需求促使大不列颠及北爱尔兰联合王国制定了总括研究协议，从而允许在预先批准的单一总体伦理和治理框架下开展多项设计相似的临床评估研究。我们对旨在支持在不同环境下对体外诊断器械以及相关检测方法的性能进行及时评估的该协议的制定、实施和演变情况进行了详细描述。总括协议允许在大流行疫情期间快速启动研究，减轻了行政负担，支持向监管机构提交申请，并实现了监测用前瞻性样品采集。尽管所描述系统反映了英国的治理结构，但是支持该方法的各项原则【包括相称性（确保监督要求与风险水平相符）、标准化以及预先批准的灵活性】适用于多种环境。该协议现已成为联合王国更广泛的大流行疫情应对准备架构的组成部分，并阐明了预先批准的可调整研究框架在疫情爆发期间是如何加速证据生成的。全世界目前都在评估从 COVID-19 大流行疫情中汲取的经验教训，且此时正是探讨研究系统是如何支持诸如总括协议之类的创新设计的大好时机。因此，我们总结了汲取的经验教训以及切合实际的考量因素，以支持其他力求在其自有的伦理和监管系统内采纳类似方法的国家。

## Резюме

Зонтичные протоколы в последнее время нашли более широкое применение в дизайне клинических исследований. Однако ценность таких протоколов для общественного здравоохранения признана пока еще не столь широко. Пандемия коронавирусной инфекции 2019 года (COVID-19) подчеркнула потребность в быстрой, надежной и масштабируемой оценке диагностических технологий. В Соединенном Королевстве Великобритании и Северной Ирландии данная потребность привела к разработке зонтичного исследовательского протокола, позволяющего проводить серию исследований по клинической оценке со схожим дизайном в рамках единой, заранее одобренной системы этического и регуляторного контроля. Авторы описывают разработку, внедрение и эволюцию данного протокола, предназначенного для своевременной оценки характеристик устройств для диагностики in vitro и соответствующих подходов к проведению исследований в различных условиях. Применение зонтичного протокола позволило быстро инициировать исследования во время пандемии, снизить административную нагрузку, оптимизировать процесс подачи документов в регулирующие органы и обеспечить проспективный сбор образцов для эпидемиологического надзора. Хотя описанная система отражает специфику британской системы управления, фундаментальные принципы данного подхода, включая пропорциональность (обеспечение соответствия требований контроля уровню риска), стандартизацию и заранее одобренную гибкость, применимы во многих странах. В настоящее время данный протокол является частью более широкой системы обеспечения готовности Соединенного Королевства Великобритании и Северной Ирландии к пандемиям и демонстрирует, каким образом заранее утвержденные адаптивные исследовательские рамки могут ускорить получение доказательных данных в условиях вспышек заболеваний. На фоне переосмысления уроков пандемии COVID-19 для мирового сообщества настало время подумать о том, каким образом исследовательские системы могут поддерживать инновационные способы организации исследований, такие как зонтичные протоколы. В связи с этим в статье обобщены полученные выводы и практические соображения с целью оказания поддержки другим странам, стремящимся внедрить аналогичные подходы в рамках своих национальных этических и регуляторных систем.

## Introduction

Robust scientific ethics and governance processes are essential for accountability and building public trust in research involving human participants, including the clinical evaluation of diagnostics.^1^ However, administrative requirements can be laborious and time-consuming.^2^ Therefore, identifying proportionate efficiencies (streamlined processes which maintain appropriate ethical oversight) is desirable when possible, particularly where research is subject to serious time constraints such as during outbreaks of infectious disease or when resources are constrained.

Umbrella protocols provide a platform to increase the efficiency of setting up evaluations of multiple investigations of treatments, schedules or devices, where protocols may otherwise be very similar. In the context of single diseases, these protocols are referred to as master protocols and are widely accepted in the context of clinical trials.^3^ The main advantage of such protocols is the move away from the typical research model that requires separate ethics and governance approvals for each study. The coronavirus disease 2019 (COVID-19) pandemic prompted an increase in interest in umbrella study designs, with a rapid review identifying 58 COVID-19 trials registered between 2020 and early 2021.^4^ Another advantage of umbrella protocols is that preapproved protocols can remain inactive for long periods of time and only be activated once required: such studies require a multiyear ethical approval and are sometimes referred to as sleeper studies.^5^ Similar approaches to pandemic preparedness are also emerging internationally; for example, the first few X cases and contacts diagnostic test evaluation contacts (FFX–Dx) protocol of the World Health Organization (WHO)^6^ and its accompanying article^7^ describe preapproved sleeper-style protocols designed to be activated rapidly during outbreaks. This development reflects a broader global interest in adaptable, ready-to-use evaluation frameworks. The FFX-Dx protocol is part of the WHO Unity Studies^8^ and is designed for the earliest stage of an outbreak to enable validation of the first molecular test for a novel pathogen.

The COVID-19 pandemic highlighted the importance of rapid, reliable diagnostic tests and techniques in the management of infectious disease outbreaks.^9^^,^^10^ In March 2020, WHO issued a protocol for the early investigation of household transmission of severe acute respiratory syndrome coronavirus 2 (SARS-CoV-2) that emphasized the importance of laboratory testing^11^ and sought to support the development of validated serological and virological assays. When a new virus emerges, speed is essential, and validating and scaling-up tests are important rate-limiting steps to slow the transmission and spread of the virus, particularly in the early phases of such a crisis.^12^

A key component of the response to the COVID-19 pandemic was the development of rapid point-of-care in vitro diagnostic devices, such as lateral flow assays, for testing asymptomatic individuals.^13^^,^^14^ New lateral flow assays were quickly developed by a wide variety of manufacturers under expedited emergency regulations.^9^^,^^14^ However, concerns soon arose about the sensitivity of these assays and regulations governing acceptable diagnostic performance.^15^^,^^16^

The challenges that arose with the COVID-19 pandemic highlighted the need for expedited and rigorous evaluation of technologies, as well as the administrative barriers associated with such evaluations.^17^^–^^19^ This need prompted the development of evaluation protocols to assess the performance of SARS-CoV-2 testing technologies in various settings (such as drive-through testing sites). These protocols were developed iteratively in response to the emergence of new testing methods and evolving circumstances.^20^^,^^21^ Considerable overlap in these protocols was observed, for instance, obtaining participant consent. The value of developing ethically preapproved umbrella protocols was therefore recognized as a way to expedite research and improve proportionate efficiencies.

## Approach

In response to challenges during the COVID-19 pandemic, we describe the development and implementation of an umbrella protocol by the Public Health and Clinical Oversight Directorate within the National Health Service (NHS) Test and Trace programme in the United Kingdom of Great Britain and Northern Ireland. The aim was to provide an overarching ethics and governance framework for sub-studies that enabled the rapid, rigorous and standardized clinical evaluation of the performance of in vitro diagnostic devices or testing practices (including sampling methods and service designs) in the United Kingdom, while recognizing that these devices can vary substantially in their technical characteristics, intended use and performance requirements, even for the same disease.

### Aims

We aimed to develop a standardized umbrella research protocol for conducting studies to obtain new evidence on the clinical performance of testing technologies, methods and testing intervals (the required frequency of repeated testing for an individual) in various settings. This protocol needed to ensure that ethics, governance, scientific and public health research standards were consistently maintained, while providing flexibility to add new diagnostic tests, interventions and/or approaches.

The protocol was designed to cover: (i) studies evaluating a different testing option (such as a new device) within a standardized process; and (ii) studies evaluating the use of a standardized test in a different process or context, for example, a new sampling technique or a new pathogen variant of concern.

The primary outcome measures were determination of diagnostic accuracy standards, that is, sensitivity, specificity and invalid rate (internal control failure), when used in various settings. Secondary outcomes included sensitivity stratified by covariates such as viral concentration, symptom status, symptom duration, vaccination status, variant status and demographic characteristics (e.g. age, sex, region and ethnicity), together with usability validation (assessment of users’ ability to correctly and safely use the device). Under the umbrella protocol, evaluation-specific ethics and governance approvals no longer needed to be obtained prospectively for studies falling within its scope. Instead, only study-specific sub-protocols and statistical analysis plans were required, as the overarching framework had already been approved.

While the protocol originally focused on SARS-CoV-2, the method is also applicable to studies addressing any infectious agent. Participants in future studies may be individuals known or suspected of having any infectious disease that comes under a National Testing or Surveillance Programme of the United Kingdom Health Security Agency or equivalent health protection initiative, contacts of such individuals, or members of at-risk groups such as health workers or hospital inpatients. In addition to COVID-19, the umbrella protocol has also been used for evaluations of influenza^22^ and group A streptococcal lateral flow assays (ongoing study).

### Development of the protocol

An overview of the development and evolution of the umbrella protocol is provided in Table 1 and summarizes the key approval dates and revisions from 2021 onwards. The main objective was to support the rapid deployment of new testing methods using a variety of study designs under a common protocol. However, other factors also contributed to the evolution of the umbrella protocol (Box 1). These factors included the use of studies to generate evidence to support submissions to regulatory bodies, inform policy decision-making and support ongoing surveillance efforts.

**Table 1:** Timeline of key steps in the evolution of the umbrella protocol for the clinical evaluation of diagnostics in infectious disease, United Kingdom of Great Britain and Northern Ireland, 2021–2024

Date	Step	Description
25 March 2021	Department of Health and Social Care submission of the umbrella protocol to Public Health England Research Ethics and Governance Group for evaluation	Early protocol development: standalone evaluations consolidated
20 April 2021	Approval of the umbrella evaluation submission by Public Health England Research Ethics and Governance Group (R&D 438)	Enabled consistent evaluation of multiple lateral flow assays
11 May 2021	Approval of amendment to accommodate evaluation of home testing using a lateral flow assay and submission of a sample for PCR	Allowed iterative expansion to home testing, pharmacy settings and new devices, refined sampling workflows and data governance
4 November 2021	Approval of amendments for rapid home testing and pharmacy evaluation	
31 December 2021	Approval of an amendment for evaluation of a self-test lateral flow assay to detect the Omicron variant of SARS-CoV-2	
7 January 2022	Approval of an amendment for evaluation of a lateral flow assay in Scotland	Not applicable
2 March 2022	Approval of an amendment (version 1.09) for evaluation of a double PCR self-test^a^	Continued refinement of study designs
27 September 2022	NHS Research Ethics Committee approval of UKHSA IVDD Clinical Evaluation Framework Protocol (v1; R&D 515)	Transitioned to UKHSA umbrella research protocol; scope expanded to NHS and adult social care settings; 5-year approval established applicable to SARS-CoV-2 and future infectious diseases
15 November 2022	NHS Research Ethics Committee approval of UKHSA IVDD Clinical Evaluation Framework Protocol (v2; R&D 515)	Minor amendments requested by the ethics committee as part of the approval
16 February 2023	NHS Research Ethics Committee approval of UKHSA IVDD Clinical Evaluation Framework Protocol (v3; R&D 515)	Changes made to include provision for surveillance activities including sample collection for serology
4 October 2023	NHS Research Ethics Committee approval of UKHSA IVDD Clinical Evaluation Framework Protocol (v4; R&D 515)	Changes made to include reimbursement to host institutions (e.g. adult social care homes) and addition of self-collection of blood samples using CE-marked devices
26 November 2024	NHS Research Ethics Committee approval of UKHSA IVDD Clinical Evaluation Framework Protocol (v5; R&D 515)	Changes made to include use of unlinked anonymized residual diagnostic samples for validation or development of new testing methods in the NHS

CE: *Conformité Européenne*; NHS: National Health Service; PCR: polymerase chain reaction; R&D: research and development; SARS-CoV-2: severe acute respiratory syndrome coronavirus 2; UKHSA IVDD: United Kingdom Health Security Agency in vitro diagnostic device.

^a^ Updated protocol version 1.09, dated 8 March 2022.

Box 1 **Factors driving the development and evolution of the umbrella protocol for clinical evaluation of diagnostics in infectious disease, United Kingdom of Great Britain and Northern Ireland**

***Public health needs***

- Reducing pathogen transmission
- Ensuring test accuracy and validity
- Ensuring suitability of testing services
- Protecting vulnerable populations
- Supporting service innovation

***Evidence generation needs***

- Assuring testing services
- Informing public health decision-making
- Supporting ongoing surveillance

***Regulatory requirements***

- Upholding research ethics
- Protecting data
- Supporting post-market surveillance
- Supporting applications and evidence submissions to regulatory authorities

***Forward-looking considerations***

- Learning from the coronavirus disease 2019 experience
- Supporting pandemic preparedness

The protocol also permitted the collection of additional samples for diagnostic evaluation, allowing repeat sampling to be undertaken specifically to support test development and validation. The protocol was designed to provide flexibility, which enabled study parameters to be rapidly adjusted and tailored via sub-study protocols to fulfil future evidence needs of the United Kingdom Health Security Agency. The new evidence needed could be, for example, about new test technologies, test performance in new settings, or performance of new testing approaches or schedules in light of new variants of concern.

On 28 September 2022, the protocol was submitted for centralized ethical and governance review (a streamlined process via the Health Research Authority) allowing participating NHS sites to avoid duplicating these checks locally. The nature of the umbrella protocol was explained clearly during its submission for approval.

This protocol was developed in accordance with relevant regulations and guidelines, including the Declaration of Helsinki,^23^ the Data Protection Act 2018,^24^ the UK Policy Framework on Health and Social Care Research,^25^ and legislation and guidelines specific to the approval of in vitro diagnostic devices.^26^^,^^27^ The UK Policy Framework on Health and Social Care Research allows evidence from regulatory-compliant sub-studies to be submitted to the Medicines and Healthcare products Regulatory Agency, which is the United Kingdom’s regulator for the pharmaceutical and medical device industries, in support of emergency use authorizations (where an urgent public health need exists)^28^ and applications for conformity markings. To meet regulatory requirements, the sub-study protocol must be submitted to the regulator at least 60 days before starting the investigation to ensure sufficient time for regulatory review and approval. In practice, manufacturers wishing to generate regulatory-compliant evidence engage with the United Kingdom Health Security Agency to define sub-study requirements. The sub-study protocol and statistical analysis plan undergo independent review and, if approved, are then conducted under the umbrella approval, ensuring that resulting data meet the requirements of the Medicines and Healthcare products Regulatory Agency.

Additionally, the umbrella protocol supports the prospective collection of samples for surveillance measures (testing to monitor infection trends), including viral load analysis and genomic sequencing.

### Specifications for the protocol

The umbrella protocol provides the single overarching ethics and governance approval, and the ethics committee does not review each activated sub-study protocol.

In practice, to give the framework sufficient scope and flexibility, while enabling ethical reassurances, study components may be grouped in three broad ways. First, elements that do not differ between sub-studies and are defined primarily within the overarching umbrella protocol. These elements may include governance arrangements such as data protection, indemnity and insurance, and cyber security. Second, elements that may differ between sub-studies but that will be selected from a preapproved long list when creating the sub-study protocol. These elements may include components of the study design and conduct such as sample collection methods and processes for obtaining and documenting consent. Third, elements that differ between sub-studies and will be independently reviewed by members of the scientific advisory committee as part of the sub-study protocol and statistical analysis plan. These elements may include statistical elements such the sample size and power calculations.

An overview of the key areas covered by the umbrella protocol and how they are categorized according to the above outline is provided in Box 2. Detailed specifications in a tabular form and the current version of the umbrella protocol are available in the online repository.^29^

Box 2 **Key elements of the umbrella protocol informing the design and conduct of sub-studies, United Kingdom of Great Britain and Northern Ireland**

***Research governance and data management***

- Prospective reviews and peer reviews (defined in umbrella protocol)
- Handling of protocol deviations or breaches (defined in umbrella protocol)
- Data protection (defined in umbrella protocol)
- Ethics and regulatory standards (defined in umbrella protocol)
- Funding and reimbursement (defined in umbrella protocol)
- Publication of study outputs (defined in umbrella protocol)
- Archiving of study data (defined in umbrella protocol)
- Indemnity and insurance (defined in umbrella protocol)
- Cybersecurity arrangements (defined in umbrella protocol)
- Assessment of participant capacity (defined in umbrella protocol)

***Study design and conduct***

- Choice of in vitro diagnostic device and testing procedure (defined in sub-study protocol)
- Participant inclusion and exclusion criteria (all available criteria set out in the umbrella protocol; selected criteria defined in sub-study protocol)
- Identification and recruitment of participants (all available recruitment approaches set out in umbrella protocol; selected approaches defined in sub-study protocol)
- Obtaining and documenting of consent (all available consent procedures set out in umbrella protocol; selected consent procedures defined in sub-study protocol)
- Sample collection, processing and disposal (all available sample types set out in umbrella protocol; selected sample types defined in sub-study protocol; and sample processing and disposal defined in umbrella protocol)
- Prevention and control of infection (defined in umbrella protocol)
- Communication of test results: (i) blinding and unblinding; (ii) resolution of conflicting results, decision matrix (all available options set out in the umbrella protocol; selected options defined in sub-study protocol)
- Handling of early discontinuation or withdrawal (defined in umbrella protocol)
- Specification of end of study (defined in umbrella protocol)
- Reporting of adverse events (defined in umbrella protocol)
- Specification of quality assurance (defined in umbrella protocol)

***Statistical analysis***

- Development and review of statistical analysis plan (defined in sub-protocol and statistical analysis plan)
- Determination of sample size (defined in sub-study protocol and statistical analysis plan)
- Specification of interim analyses (defined in sub-study protocol and statistical analysis plan)
- Specification of primary and secondary outcomes (all potential outcomes set out in umbrella protocol; selected outcomes defined in sub-study protocol)

Relevant comparators for new diagnostic tests include standard, validated diagnostic tests whose results are used for clinical management. In contrast, results from the test under evaluation are not used to guide clinical care. Studies conducted under the umbrella protocol may support different clinical use cases, such as screening, diagnosis or surveillance, which have distinct ethical and operational considerations. These cases are accommodated through the sub-study protocols, which specify the intended use and associated governance requirements.

Study designs within the remit of the protocol include studies in which participants: (i) perform (or have professionally administered) several tests simultaneously or within a few hours of each other; (ii) perform tests on a regular basis for a set period (e.g. the incubation period); (iii) perform tests using different sample collection methods or materials (e.g. saliva versus nose and/or throat swab, or viral inactivation versus viral transport medium) to compare the effect of sample type or collection approach; and (iv) provide samples to be used for in vitro validation of in vitro diagnostic devices in the laboratory or for other functions that form part of the health protection response (e.g. viral characterization). Relevant sample types may include swabs (e.g. shallow nasal, throat, saliva and skin or lesion), respiratory specimens (e.g. breath condensate and sputum) and body fluids (e.g. blood, serum and urine).

The protocol incorporates procedures for obtaining participant consent and verbal agreement for handling their personal data (online repository).^29^ These procedures provide for communications with participants required under relevant regulations; for example, ensuring that participants are informed of the purposes for which their personal data will be processed, including any secondary use of these data for additional public health research purposes.

## Implementation

Since the approval of the original umbrella protocol through to the time of writing this paper, many sub-studies have been conducted under this protocol. These investigations include studies at local or regional testing sites (conducted under the first protocol: R&D 438; Table 1)^30^ and multisite evaluations in long-term care settings, known as adult social care, in the United Kingdom.

Having ethical approval in place for 5 years and recruiting sites already onboarded (i.e. sites that have completed local capacity and capability checks and any one-off setup processes, thus requiring no ongoing funding or maintenance between studies) greatly reduces the time to start and complete recruitment. This situation in turn reduces the administrative burden and cost for the research teams and health services at a time when they are under the most operational pressure. As a result, this approach may prove particularly useful in resource-constrained settings where rapid activation and efficient use of resources are essential. An overview of the studies that have used, or are currently using, the umbrella protocol is provided in Table 2.^19^^–^^21^^,^^30^

**Table 2:** Summary of studies conducted under the umbrella protocol for the clinical evaluation of diagnostics in infectious disease, United Kingdom of Great Britain and Northern Ireland, 2021–2024

Evaluation identification number	Objective	No. of participants recruited	Recruitment start date	Recruitment end date
LFD014	Evaluate effectiveness of self-swabbing of anterior nares using Innova lateral flow assay at participants’ homes	6591	23 August 2021	9 November 2021
LFD015	Evaluate effectiveness of self-swabbing of anterior nares using SureScreen lateral flow assay at participants’ homes	1534	27 July 2021	20 August 2021
LFD016	Confirm effectiveness of self-swabbing of both nostrils to mid-turbinate level using Innova lateral flow assay	1156	23 June 2021	26 July 2021
LFD017	Measure diagnostic performance metrics of lateral flow assay tests currently used within NHS test and trace for detecting the Indian variant of concern when individuals performed self-swabbing of nose and throat using Innova lateral flow assay	635	22 May 2021	25 June 2021
LFD018	Measure diagnostic performance metrics and effectiveness of assisted swabbing of anterior nares using Innova lateral flow assay	1226	30 November 2021	24 December 2021
LFD025	Assess comparability of diagnostic performance of a multiday lateral flow assay test using Acon lateral flow assay kits and a single PCR test	2792	11 August 2021	15 December 2021
LFD026	Evaluate performance of Acon self-test to detect Omicron variant of SARS-CoV-2	1910	11 January 2022	2 February 2022
LFD101–104^a^	Evaluate self-testing of symptomatic and asymptomatic participants performed with lateral flow assay kits distributed at physical sites	86 961	8 November 2020	21 March 2022
PCR004^b^	Estimate difference in average viral loads obtained from two sequential mid-turbinate nasal and throat PCR samples (quality assurance)	907	25 February 2022	15 March 2022
PCR005	Understand the relationship and relative contribution of nose and throat anatomical swabbing sites on testing performance to detect SARS-CoV-2 Omicron variant of concern, and how this relationship varies with time from symptom onset (quality assurance)	817	21 March 2022	30 March 2022
LFD031	Compare diagnostic sensitivity of two lateral flow assay kits (Innova and Orient Gene) in a care home setting	2004	30 August 2022	12 December 2022
MULTI-01	Evaluate diagnostic sensitivity of multiplex lateral flow assay to detect influenza A and B in various settings	130	27 January 2023	31 March 2023
MULTI-02 (adult social care)	Evaluate diagnostic sensitivity of multiplex lateral flow assay to detect influenza A and B in a care home setting	48	10 October 2023	22 March 2024
Guy’s and St Thomas’ Hospital	Evaluate performance (diagnostic sensitivity) of combined multiplex-lateral flow assay, testing for SARS-CoV-2 and influenza A and B, compared with PCR in a hospital emergency department	808	1 December 2022	21 April 2023

PCR: polymerase chain reaction; SARS-CoV-2: severe acute respiratory syndrome coronavirus 2.

^a^ Ongoing evaluation as of 28 May 2024.

^b^ The protocol for this study is supplied as an exemplar in the online repository.^29^

The first version of the umbrella protocol was designed to provide operational capabilities in the assessment of clinical performance of new testing technologies, methods and intervals in various settings. However, the scope of that early version did not extend to NHS settings and did not allow use in future activities beyond the COVID-19 context. Unlike the current version, the early version did not build in the flexibility to permit subsequent sub-studies to be exempted from prospective approvals by the Public Health England Research Ethics and Governance Group. The updated protocol does not have this problem as it has inbuilt flexibility.

The evolution of the umbrella protocol into a full-fledged research protocol in late 2022 enabled its scope to be expanded to cover clinical investigations of new devices and to encompass new populations including NHS sites and adult social care settings. The expanded umbrella protocol therefore required approval from the Health Research Authority and NHS Research Ethics Committee ahead of adoption by the National Institute for Health and Care Research Clinical Research Network portfolio.^31^

## Discussion

In its current form, the umbrella protocol is a key component of the United Kingdom’s pandemic preparedness.

With suitable foresight when developing an umbrella protocol, a broad scope of future sub-study designs can be accommodated within a framework, allowing adaptation to changing practical and clinical requirements. Importantly, this adaptation can be done without requiring repeated amendments to be submitted for approval, while maintaining rigorous governance. Extensive adjustments, beyond the scope set out in the approved umbrella, still require ethical approval, underlining the importance of anticipating future developments.

The United Kingdom was not alone in developing umbrella protocols. In November 2020, WHO issued a protocol for the implementation of lateral flow assays specifically in symptomatic settings (the United Kingdom protocol supports studies in both symptomatic and asymptomatic settings). The WHO protocol covers assessment of performance as well as acceptability, feasibility and impact.^32^ While the WHO protocol and United Kingdom umbrella protocol described here were both developed in the context of the COVID-19 pandemic, this concept has clear potential for much wider application. Hence, the United Kingdom protocol has the flexibility to allow studies to be conducted on any future pathogenic threats.

Additionally, the length of the ethical approval of the United Kingdom protocol (5 years) allows it to function as a so-called sleeper study, remaining inactive but ready for activation when required. This preparedness approach has been recognized as a critical learning from the experience of the COVID-19 pandemic.^12^^,^^33^ In alignment with the WHO Pandemic Agreement,^34^ this protocol will support the rapid evaluation and sharing of results of standardized studies of in vitro diagnostic devices for new pandemic pathogens. Umbrella protocols, such as that described here, provide guidance on trial design under a single overarching protocol, without the need to develop full protocols for every sub-study. Just as platform trials based on master protocols have shown significant efficiency and cost benefits within adaptive clinical trials,^35^ this umbrella protocol has the potential to do likewise for diagnostic evaluation studies.

For countries considering a similar approach, several practical steps may support the development of an umbrella protocol. These steps include identifying study elements (as highlighted in Box 2) that can be standardized within a single overarching approval, such as consent procedures, data governance requirements, sample collection and biobanking, and engaging early on with ethics, governance and regulatory bodies to determine which components may be preapproved and thus agreeing a course of action from the outset.

The umbrella protocol has several strengths and challenges. The key strength of the protocol is its flexibility and adaptability. During the COVID-19 pandemic, the protocol enabled rapid roll-out of new studies through a standardized approach to key elements such as obtaining and recording participant consent. After required approval, the protocol could be used to support studies being conducted within the NHS and adult social care settings, thereby enabling key populations to be accessed. Other strengths include the ability to make ongoing improvements, for example, refinements to sampling workflows or procedures evaluating safe and correct use of devices, within subsequent sub-study protocols without altering the approved umbrella protocol itself.

When designing standalone protocols, there is value in considering whether the use of an umbrella protocol may be beneficial, as it may allow for expedited study conduct and resource savings, support greater standardization across the design and conduct of subsequent sub-studies, and enhance the comparability of data generated across studies. Indeed, most standalone protocols could have elements of an umbrella protocol incorporated. For example, if a manufacturer wished to validate several different devices with only slight variations in each study design, a suitable umbrella protocol, designed with forethought, would enable them to undertake this range of studies with minor design variations, without requiring individual ethics approvals or governance processes for each study.

A major difficulty that could not be fully overcome with our umbrella protocol was the challenge of administrative systems that are incompatible with the protocol’s flexible operational model. For instance, the Atrributing the cost of health and social care research (ACoRD) framework has contributed substantially to accelerating study set-up by standardizing the process for cost attribution in England;^36^ however, that framework is not well suited to studies where the full range of potential study activities is unclear at the outset. Additionally, some sponsor and health-service trial management and reporting processes in the United Kingdom are not configured to accommodate sleeper protocols, leading to inappropriate flags that studies have failed to recruit participants. Organizational reluctance also existed among NHS trusts to expend effort in setting up sleeper studies that may remain dormant for an undefined period. These challenges have largely been overcome through communication and negotiation, but they highlight the need for procedural changes to modernize research systems.

In conclusion, since its introduction in the United Kingdom, the umbrella protocol has ensured that studies critical for the COVID-19 pandemic response could be delivered quickly and safely under one standardized ethics and governance framework. The protocol has built-in flexibility to continue to inform key policy decisions, support the development of improved testing approaches and provide assurance of the clinical effectiveness of United Kingdom testing programmes. While the protocol was developed in response to the demands of the COVID-19 pandemic, it has potential for application to other infectious diseases with pandemic potential, and to contribute to surveillance and biobanking efforts. This protocol therefore represents a key component of the United Kingdom’s pandemic preparedness and a possible model for the future design of evaluation studies in diagnostic tests and strategies. While different countries will have their own research ethics and research infrastructure, we have outlined the rationale and usefulness of adopting this approach as part of pandemic preparedness. We have also outlined a specific precedent that may support countries considering how umbrella protocols can be implemented as part of their pandemic preparedness activities.
